# The cost-effectiveness of a theory-based online health behaviour intervention for new university students: an economic evaluation

**DOI:** 10.1186/1471-2458-14-1011

**Published:** 2014-09-27

**Authors:** Jen Kruger, Alan Brennan, Mark Strong, Chloe Thomas, Paul Norman, Tracy Epton

**Affiliations:** School of Health and Related Research, University of Sheffield, Regent Court, 30 Regent Street, Sheffield, S1 4DA United Kingdom; Department of Psychology, University of Sheffield, Western Bank, Sheffield, S10 2TP United Kingdom

**Keywords:** Alcohol, Fruit and vegetables, Exercise, Smoking, Health behaviour, Cost-effectiveness, Economic evaluation, Economic model, Young people, Students, University, Costs

## Abstract

**Background:**

Too many young people engage in unhealthy behaviours such as eating unhealthily, being physically inactive, binge drinking and smoking. This study aimed to estimate the short-term and long-term cost-effectiveness of a theory-based online health behaviour intervention (“U@Uni”) in comparison with control in young people starting university.

**Methods:**

A costing analysis was conducted to estimate the full cost of U@Uni and the cost of U@Uni roll-out. The short-term cost-effectiveness of U@Uni was estimated using statistical analysis of 6-month cost and health-related quality of life data from the U@Uni randomised controlled trial. An economic modelling analysis combined evidence from the trial with published evidence of the effect of health behaviours on mortality risk and general population data on health behaviours, to estimate the lifetime cost-effectiveness of U@Uni in terms of incremental cost per QALY. Costs and effects were discounted at 1.5% per annum. A full probabilistic sensitivity analysis was conducted to account for uncertainty in model inputs and provide an estimate of the value of information for groups of important parameters.

**Results:**

To implement U@Uni for the randomised controlled trial was estimated to cost £292 per participant, whereas roll-out to another university was estimated to cost £19.71, both giving a QALY gain of 0.0128 per participant. The short-term (6-month) analysis suggested that U@Uni would not be cost-effective at a willingness-to-pay threshold of £20,000 per QALY (incremental cost per QALY gained = £243,926). When a lifetime horizon was adopted the results suggest that the full implementation of U@Uni is unlikely to be cost-effective, whereas the roll-out of U@Uni to another university is extremely likely to be cost-effective. The value of information analysis suggests that the most important drivers of decision uncertainty are uncertainties in the effect of U@Uni on health behaviours.

**Conclusions:**

The study provides the first estimate of the costs and cost-effectiveness of an online health behaviour intervention targeted at new university students. The results suggest that the roll-out, but not the full implementation, of U@Uni would be a cost-effective decision for the UK Department of Health, given a lifetime perspective and a willingness-to pay threshold of £20,000 per QALY.

**Trial registration:**

Current Controlled Trials ISRCTN67684181.

**Electronic supplementary material:**

The online version of this article (doi:10.1186/1471-2458-14-1011) contains supplementary material, which is available to authorized users.

## Background

Too few young people engage in healthy behaviours such as eating healthily, being physically active, consuming alcohol sensibly and not smoking. For example, evidence from the 2008 Health Survey for England [1] suggested that only 20% of young people (aged 16–24) eat five portions of fruit and vegetables per day, less than 50% meet weekly physical activity guidelines, 25% smoke, and 40% exceed daily recommended alcohol limits. These health behaviours amongst young people have an effect on health in terms of risk of illness and disease, mortality risk, and quality of life [2].

The transition from school to university offers a unique opportunity to intervene to promote the adoption of healthy behaviours in young people, as a large number of people can be targeted easily and at a time when they may be open to changes to their lifestyle [3]. An online health promotion intervention (“U@Uni”) has been designed to use the transition from school to university to promote healthy behaviours in young people [3]. The intervention was developed based on three key psychological theories: self-affirmation [4], the Theory of Planned Behaviour [5], and implementation intentions [6]. In summary, the intervention provides an online portal for young people to self-affirm, view multimedia theory-based health messages, make implementation intentions and access information about healthy resources (such as exercise facilities and greengrocers) in their local area. U@Uni is designed to be accessed by young people in the month before they start university. The intervention has been compared to a ‘measurement only’ control condition in a randomized controlled trial (RCT) of 1,445 young people beginning their studies at the University of Sheffield, United Kingdom (UK). Data were collected from RCT participants at baseline and at 1-month and 6-month follow-up. Full details of the U@Uni intervention and the RCT have been published elsewhere [3, 7].

To determine whether an intervention provides good value for money, an economic evaluation comparing the costs and benefits of the intervention with those of a relevant control is required. In the UK, the National Institute for Health and Care Excellence (NICE) is the organization responsible for evaluating health technologies and producing clinical and public health guidelines. NICE recommends that public health interventions be evaluated using quality-adjusted life years (QALYs) as the measure of benefit [8]. QALYs are a combined measure of length and quality of life, and are calculated by multiplying the number of years spent in a particular state of health by a utility value for that health state, derived from a preference-based measure of health-related quality of life [9]. This study aimed to estimate the short-term (6-month time horizon) and long-term (lifetime time horizon) cost-effectiveness of the U@Uni online health behaviour intervention in young people starting university. Given the absence of current best practice guidance, the intervention was compared solely against the ‘measurement only’ control condition from the RCT.

## Methods

The economic evaluation was conducted in three parts: (i) a costing analysis to estimate the cost of U@Uni, (ii) a within-trial statistical analysis to estimate the short-term cost-effectiveness of U@Uni using EQ-5D data [10] collected within the 6-month RCT follow-up period, and (iii) an economic modelling analysis to estimate the long-term cost-effectiveness of U@Uni. Economic modelling was necessary to extrapolate the health behaviour outcomes from the RCT over the long term and to link the intermediate health behaviour outcomes with long-term costs, life years and QALYs. Economic modelling provides the necessary framework for synthesis of data from multiple sources in order to estimate long-term cost-effectiveness. In this study individual-level 6-month follow-up data from the U@Uni RCT (N = 1,445) [7] were synthesized with individual-level cross-sectional data on health behaviours in the general UK population from the Health Survey for England 2008 (N = 14,925) [1] and hazard ratios linking all four of the health behaviours to mortality risk from a published survival analysis (N = 4,886) [11]. The perspective of the study is that of the UK Department of Health. Ethical approval for the RCT was obtained from the Department of Psychology Research Ethics Committee at the University of Sheffield.

### Costing analysis

The cost of U@Uni was estimated using a questionnaire administered to the staff involved in the development and implementation of the intervention from the Department of Psychology and the Department of Computer Science at the University of Sheffield. Each member of staff was asked to estimate the number of hours they had spent on different aspects of developing and implementing U@Uni (central estimate and upper and lower bounds to represent their uncertainty in their central estimates). Examples of aspects that Department of Psychology members of staff were asked to estimate included “developing health messages” and “development of local elements” (local elements included information about healthy resources available in the local area e.g. exercise facilities and greengrocers). Examples of aspects that Department of Computer Science members of staff were asked to estimate included “developing the website version” and “website monitoring and maintenance”. See section A of Additional file 1 for further details. The University of Sheffield’s University Research Management System was used to estimate the full economic cost of all staff time using individual staff members’ salaries and working hours plus additions for on-costs (i.e., tax, national insurance and pensions) and overheads. Non-staff costs included the cost of questionnaire software and payments to participants for formative research used to develop the health messages. Two different per-person costs of U@Uni were calculated:

First, the within-trial cost of full development and implementation of the intervention at the University of Sheffield was estimated:


Second, the roll-out cost of implementing the intervention at other UK universities was estimated, assuming that University of Sheffield salaries and overheads are nationally representative: [12]


Means and upper and lower 95% confidence intervals for the mean population-level cost for each of the two cost scenarios were generated (see section A of Additional file 1 for further details).

### Within-trial cost-effectiveness analysis (short-term 6-month time horizon)

The baseline characteristics of participants in the RCT are presented in Table 1. The full development and implementation cost of the U@Uni intervention and the cost of other health care resources consumed during the 6-month follow-up period of the U@Uni RCT were weighed against the QALYs accrued during this time in a within-trial cost-effectiveness analysis. The cost of the intervention was estimated as outlined above. The healthcare resources used by individuals in each arm of the RCT were estimated using a questionnaire given to participants at 6-month follow-up that asked them to report the number of GP visits, hospital inpatient admissions, hospital outpatient attendances, emergency department attendances and ambulance call-outs they had needed during the previous 6 months. Unit costs were attached to each of these resources using NHS Reference Costs 2011-12 [13] and the Unit Costs of Health and Social Care 2012 [14] (see section B of Additional file 1 for further details). The QALYs accrued by participants in each arm of the RCT were estimated from health-related quality-of-life data measured using preference-based EQ-5D utility values [10] collected at baseline, 1-month and 6-month follow-up. Missing utility data at either follow-up point was imputed with that individuals’ utility value from the other follow-up point (a combination of last observation carried forward and last observation carried backward). QALYs were calculated using the trapezium rule [15].

**Table 1:** **Model input parameters**

Parameter	Distribution	Parameters*	Source
**Individual characteristics**			
Age (years)	Individual-level data	Mean = 18.90	U@Uni RCT
		SD = 2.49	
Gender	Individual-level data	42% Male	
		58% Female	
Fruit and vegetables (portions per day)	Individual-level data	Mean = 6.52	
		SD = 4.94	
Alcohol (units per week)	Individual-level data	Mean = 11.51	
		SD = 18.62	
Physical activity (minutes per week)	Individual-level data	Mean = 163.35	
		SD = 121.74	
Smoking status	Individual-level data	12% Smoker	
		88% Non-smoker	
**Costs**			
U@Uni cost (full development)	Lognormal	Mean ln(cost) = 12.2763	Costing analysis
		SD ln(cost) = 0.0513	
U@Uni cost (roll-out)	Lognormal	Mean ln(cost) = 10.3349	Costing analysis
		SD ln(cost) = 0.0663	
**Intervention effect regression coefficients**			
β_0_ Fruit and vegetables: constant	Multivariate normal (see section C of Additional file 1 for covariance matrix)	Mean = 3.0095	U@Uni RCT
β_1_ Fruit and vegetables: baseline behaviour coefficient		Mean = 0.2482	
β_2_ Fruit and vegetables: age coefficient		Mean = 0.0586	
β_3_ Fruit and vegetables: gender coefficient (1 = male; 0 = female)		Mean = -0.0252	
β_4_ Fruit and vegetables: intervention coefficient (i.e. mean effect of U@Uni on portions of fruit and vegetables per day compared to control)		Mean = -0.1116	
ϵ Fruit and vegetables: residual	Normal	Mean = -3.18 × 10^-09^	U@Uni RCT
		SD = 4.8973	
β_0_ Alcohol: constant	Multivariate normal (see section C of Additional file 1 for covariance matrix)	Mean = 18.9219	U@Uni RCT
β_1_ Alcohol: baseline behaviour coefficient		Mean = 0.4834	
β_2_ Alcohol: age coefficient		Mean = -0.5772	
β_3_ Alcohol: gender coefficient (1 = male; 0 = female)		Mean = -0.4252	
β_4_ Alcohol: intervention coefficient (i.e. mean effect of U@Uni on units of alcohol per week compared to control)		Mean = -0.4275	
ϵ Alcohol: residual	Normal	Mean = 1.85 × 10^-08^	U@Uni RCT
		SD = 19.9055	
β_0_ Physical activity: constant	Multivariate normal (see section C of Additional file 1 for covariance matrix)	Mean = 161.3326	U@Uni RCT
β_1_Physical activity: baseline behaviour coefficient		Mean = 0.2339	
β_2_ Physical activity: age coefficient		Mean = -2.7763	
β_3_ Physical activity: gender coefficient (1 = male; 0 = female)		Mean = -2.0189	
β_4_ Physical activity: intervention coefficient (i.e. mean effect of U@Uni on minutes of physical activity per week compared to control)		Mean = 4.0141	
ϵ Physical activity: individual residual	Normal	Mean = -1.38 × 10^-07^	U@Uni RCT
		SD = 106.0016	
Probability smokers quit smoking (U@Uni)	Beta	α = 27	U@Uni RCT
		β = 33	
Probability non-smokers start smoking (U@Uni)	Beta	α = 14	U@Uni RCT
		β = 466	
Probability smokers quit smoking (do nothing)	Beta	α = 19	U@Uni RCT
		β = 45	
Probability non-smokers start smoking (do nothing)	Beta	α = 27	U@Uni RCT
		β = 462	
**Lag effects (years until full effect of behaviour change on mortality risk)**			
Fruit and vegetables lag	Lognormal	Mean = 2.7438	Expert elicitation
		SD = 0.1247	
Alcohol lag	Gamma	α = 1.3541	Expert elicitation
		β = 0.6537	
Physical activity lag	Normal	Mean = 5.5000	Expert elicitation
		SD = 1.4642	
Smoking lag	Normal	Mean = 5.5000	Expert elicitation
		SD = 1.1110	
Distribution of individual-level duration of U@Uni behavioural effect (years)**			
Mean duration	Beta	α = 1.8179	Expert elicitation
		β = 0.1304	
		Scale = 4.5000	
Standard deviation of duration	Beta	α = 2.9109	Expert elicitation
		β = 0.2691	
		Scale = 3.3800	
**Hazard ratios for effect of health behaviours on mortality risk**			
Fruit and vegetable consumption	Lognormal	Mean = 0.0953	Kvaavik et al. (2010) [11]
		SD = 0.0673	
Alcohol consumption	Lognormal	Mean = 0.1655	Kvaavik et al. (2010) [11]
		SD = 0.0840	
Physical activity	Lognormal	Mean = 0.3577	Kvaavik et al. (2010) [11]
		SD = 0.0641	
Smoking status	Lognormal	Mean = 0.3577	Kvaavik et al. (2010) [11]
		SD = 0.0873	
**Utility ordinary least squares regression model coefficients**			
β_0_ Constant	Multivariate normal (see section F of Additional file 1 for covariance matrix)	Mean = 0.9490	Analysis of Health Survey for England 2008 [1]
β_1_ Age coefficient		Mean = -0.0038	
β_2_ Gender (1 = male; 0 = female) coefficient		Mean = 0.0142	
β_3_ Fruit and vegetables (portions per day) coefficient		Mean = 0.0207	
β_4_ Alcohol (units per week) coefficient		Mean = 0.0016	
β_5_ Smoke (smoker = 1; non-smoker = 2) coefficient		Mean = -0.0541	
β_6_ Physical activity (minutes per week) coefficient		Mean = 0.0002	
β_7_ Age^2^		Mean = -4.31 × 10^-06^	
β_8_ Fruit and vegetables^2^		Mean = -0.0033	
β_9_ Fruit and vegetables^3^		Mean = 0.0001	
β_10_ Alcohol^2^		Mean = -2.77 × 10^-05^	
β_11_ Alcohol^3^		Mean = 6.45 × 10^-08^	
β_12_ Physical activity^2^		Mean = -2.59 × 10^-07^	
β_13_ Physical activity^3^		Mean = 4.88 × 10^-11^	
β_14_ Age*Fruit and vegetables β_0_ interaction		Mean = 4.94 × 10^-05^	
β_15_ Age*Alcohol interaction		Mean = 1.61 × 10^-05^	
β_16_ Age*Physical activity		Mean = 2.47 × 10^-06^	

*SD standard deviation.

**The sampled mean and standard deviation from the beta distributions are then converted to log (mean) and log (standard deviation) and used as parameters for the lognormal distribution for individual-level durations of response.

Rather than comparing the mean costs and QALYs from the raw data, regression models were used to control for differences at baseline between the two RCT arms. Ordinary least squares regression was used to estimate the effect of the RCT arm on total RCT costs (intervention cost + other healthcare costs) and total RCT QALYs. There were two regression models: One of total costs (N = 672) and one of total QALYs (N = 643), both with RCT arm as an index covariate. Age, gender, baseline fruit and vegetable consumption, baseline alcohol consumption, baseline physical activity and baseline smoking status were included as covariates in both the cost and QALY regression models. For the regression of QALYs, baseline utility value was also included as a covariate to reduce bias [16]. To examine uncertainty, non-parametric bootstrapping (5,000 replicates) was used to generate a joint distribution of mean costs and QALYs from the predicted individual-level costs and QALYs. This distribution was used to calculate the mean costs and QALYs in each arm of the trial, the incremental cost-effectiveness ratio (ICER; incremental cost per QALY), and the probability of U@Uni being cost-effective within 6 months at different willingness-to-pay thresholds.

### Long-term economic modelling (lifetime time horizon)

**Figure 1:**
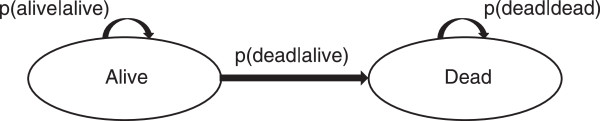
**The U@Uni long-term cost-effectiveness model - a two states Markov model.** p(dead|alive) = probability of dying if alive = a function of age, gender, fruit and vegetable consumption, alcohol consumption, physical activity and smoking status. p(alive|alive) = probability of staying alive if alive = 1 - p(dead|alive). p(dead|dead) = probability of staying dead if dead = 1.

The long-term cost-effectiveness of U@Uni was estimated using an economic model developed to translate changes in health behaviours into long-term life years and QALYs. The model structure was informed by a brief review of existing public health models that incorporated one or more of the relevant behavioural changes. It was designed as a population-based individual patient-level simulation model with annual time cycles simulating transitions between two Markov states (alive and dead) over time (see Figure 1). The baseline age, gender, daily portions of fruit and vegetables consumed, weekly units of alcohol consumed, weekly minutes of physical activity undertaken and smoking status (smoker or non-smoker) of simulated individuals were taken from baseline data collected in the U@Uni RCT (see Table 1). The same individuals were simulated under the U@Uni intervention and control conditions.

Follow-up health behaviours were modelled as a function of baseline health behaviours and U@Uni intervention effects. The effects of U@Uni on daily portions of fruit and vegetables, weekly units of alcohol and weekly minutes of physical activity were modelled using ordinary least squares regressions with age, gender and the corresponding baseline behaviour as covariates (see section C of Additional file 1). The coefficients from the regressions are presented in the ‘Intervention effect regression coefficients’ section of Table 1. Random samples from the distributions of regression residuals from each regression model were added to each individual’s simulated follow-up behaviours to represent heterogeneity in the behavioural response to U@Uni, so that the simulated value for an individuals’ follow-up behaviour was calculated as:
1

Where:

BH_T1_ = behaviour at 12 months

BH_T0_ = behaviour at baseline

age = age in years

gender = 1 if male and = 0 if female

intervention = 1 if received U@Uni and = 0 if received control

β_0_ - β_4_ = coefficients as per Table 1

ϵ = randomly sampled individual residual from distributions given in Table 1

The effects of U@Uni on smoking were modelled as a probability of quitting smoking for baseline smokers and a probability of starting smoking for non-smokers (see Table 1).

The trajectories of fruit and vegetable consumption, alcohol consumption and physical activity over time were modelled by placing each individual on an age-specific percentile rank from general population data collected as part of the Health Survey for England 2008 [1] and then keeping individuals on the same percentile throughout their lifetime under the control condition. Under the intervention condition individuals were kept on the same percentile rank for the three continuous behaviours until the duration of treatment effect was reached, at which point they reverted to the percentile rank they would have been on under the control condition. A probability distribution for the duration of treatment effect was elicited from psychological experts (see section D of Additional file 1) and each simulated individual had a different randomly sampled duration of treatment effect. Under the intervention condition the behavioural effect of U@Uni was modelled with a linear decay over the period of treatment effect. Smoking status was modelled as constant over an individuals’ lifetime under the control condition. Under the intervention condition smoking status was modelled as constant until the duration of treatment effect was reached, at which point smoking status reverted to what it would have been under the control condition.

The underlying age- and gender-specific annual probabilities of dying were taken from UK life tables [17] with the probability of dying aged 100 years or over set to 1. The annual probabilities of dying were adjusted for individual-level health behaviours using hazard ratios calculated using linear and logarithmic continuous risk functions developed from hazard ratios reported in a published survival analysis [11] weighted by health behaviours from the Health Survey for England 2008 [1] (see section E of Additional file 1 for further details). The distributions used to represent the source hazard ratios for each health behaviour are presented in the ‘Hazard ratios for effect of health behaviours on mortality risk’ section of Table 1. The hazard ratios for each simulated individual were modelled as a function of their health behaviours, e.g., a person drinking more alcohol would have a higher hazard ratio than an individual drinking less alcohol and their adjusted mortality risk would therefore also be higher.

There was assumed to be a time lag to the full effect of behaviours on mortality risk. The lags for each of the four health behaviours were elicited from epidemiological and economic modelling experts (see section D of Additional file 1) and were applied so that the full hazard ratio effect was applied after the simulated lag (with reduced hazard ratio effects applied in the preceding time cycles).

The annual probabilities of dying were used to estimate the number of life years each individual accrued over their lifetime by multiplying the number of life years accrued in the previous year by the annual probability of dying in the current year.

An ordinary least squares regression of Health Survey for England 2008 data [1] was used to construct a regression equation to predict EQ-5D utilities from fruit and vegetable consumption, alcohol consumption, smoking status, physical activity level, age and gender (see section F of Additional file 1 for further details). The coefficients from the regression are presented in the ‘Utility ordinary least squares regression model coefficients’ section of Table 1, and the equation to simulate an individuals’ utility value in any given year was:
2

Where:

U = EQ-5D utility score

age = Age in years

gender = 1 if male and = 0 if female

fv = portions of fruit and vegetables consumed per day

alc = units of alcohol consumed per week

sm = 1 if smoker and = 0 if non-smoker

pa = minutes of physical activity per week

β_0_ - β_16_ = coefficients as per Table 1

The EQ-5D utility regression equation was used to weight the life years accrued by each simulated person in each year (based on their health behaviours in that year) to generate QALYs.

Intervention costs were based on the costing analysis described above and as they occurred in the first year they were not discounted. No other costs were included in the analysis; this was considered to be a conservative structural assumption as improvements in health behaviours as a result of U@Uni could result in reduced morbidity and therefore reduced healthcare costs.

The model was fully probabilistic with each input parameter represented by a probability distribution to characterize uncertainty in the parameter values. The parameter uncertainty was propagated through the model to generate a distribution of lifetime costs and QALYs for the control and U@Uni conditions. Full details of the model parameters are given in Table 1. The economic model used a number of assumptions aside from those described above and in Table 1; these are presented in Table 2.

**Table 2:** **Model assumptions**

Assumption	Implication for modelling
6-month behaviours from the trial are assumed to represent year 1 behaviours in the model.	May over-estimate the duration of U@Uni behavioural treatment effects by 6 months.
Health Survey for England complete case data represents behavioural patterns in the general population that is represented in the ONS Life Tables.	Complete cases from Health Survey for England may be atypical and not representative of the general population in some way (unclear effect on results)
Probability of death aged >100 years = 1	May under-estimate life expectancy increases as a result of U@Uni.
Assumes health behaviours from Health Survey for England when aged over 90 are equal to those when aged 90	Health behaviours of people aged over 90 may be different from those of people aged 90 in some way (unclear effect on results).
Assumes relationship between alcohol and mortality risk is linear and between physical activity or fruit and vegetables and mortality risk is logarithmic (to avoid negative values for hazard ratios)	The shape of the true relationship between the health behaviours and mortality risk may have a different functional form (unclear effect on results).
Hazard ratio for 0 portions fruit and vegetables = 1.6	May under- or over-estimate the increased mortality risk associated with eating no fruit and vegetables (unclear effect on results).
Hazard ratio for 0 minutes of physical activity = 1.6	May under- or over-estimate the increased mortality risk associated with doing no physical activity (unclear effect on results).
Except for due to the effect of U@Uni an individuals’ fruit and vegetable consumption, physical activity, and alcohol consumption measured 6-months after university are assumed to stay on the same age-specific percentile rank from the general population throughout their lifetime	Individuals’ health behaviours could be expected to vary more than this over a lifetime (unclear effect on results).
Except for due to the effect of U@Uni an individuals’ smoking status measured 6-months after university is assumed to stay fixed throughout their lifetime	Individuals’ smoking status could be expected to vary more than this over a lifetime (unclear effect on results).
Health behaviour change decays linearly up to the year of the maximum length of the treatment effect	Behaviour change may decay non-linearly (unclear effect on results).
Hazard ratios for the effect of health behaviours on mortality risk are age independent	The relative effect of health behaviours on mortality risk may vary with age (unclear effect on results).

### Economic analysis

The model was used to evaluate the cost-effectiveness of the full development and implementation of U@Uni as per the process at the University of Sheffield and the cost-effectiveness of rolling out U@Uni to other universities. Life years and QALYs were discounted annually at 1.5% as recommended for public health economic evaluations by NICE [8]. In each scenario the lifetime costs and QALYs of U@Uni were compared to those of the control condition. In addition, a threshold analysis was conducted to identify the threshold price per person that would be required for U@Uni to be considered cost-effective at the NICE willingness-to-pay threshold of £20,000 per QALY [8].

### Uncertainty

A full probabilistic sensitivity analysis was conducted to estimate the probability of U@Uni being cost-effective at different willingness-to-pay thresholds. Monte Carlo probabilistic sensitivity analysis was conducted by randomly sampling a value for each input parameter, running the model, and repeating this process 5,000 times. For each run 1,000 individuals were simulated. The probabilistic sensitivity analysis was used to generate a joint distribution of incremental costs and QALYs, jack-knife confidence intervals for the ICER, and a cost-effectiveness acceptability curve [18]. The expected value of collecting further data to populate model parameters was estimated using expected value of perfect information analysis [19, 20] with a focus on identifying which parameters should be targeted in any further research [21]. Three structural sensitivity analyses were conducted to explore the sensitivity of the model results. First, a different discount rate for costs and QALYs (3.5% per annum as recommended by the NICE methods guide for health technology appraisal [22]); second, the use of a utility regression function that used only age and gender as predictors and therefore did not account for the effects of health behaviours on quality of life [23] and third, varying the duration of intervention effect from 4.5 years to between one and ten years.

## Results

### Costing analysis results

The results of the costing analysis suggested that U@Uni would cost £208,500 (95% confidence interval £194,723 to £238,611) to fully develop and implement as per the University of Sheffield U@Uni RCT. This included £119,179 in staff costs, £2,355 in non-staff costs (for questionnaire software and payments to participants in formative research), and £86,966 in overheads (including estates, infrastructure and indirect costs). Based on the number of participants in the intervention arm of the U@Uni RCT (N = 736) the per-person cost to fully develop and implement U@Uni was estimated to be £283.29. It was estimated that rolling out U@Uni to another university would cost an average of £29,988 (95% confidence interval £27,054 to £35,081). This included £13,146 in staff costs, £300 in non-staff costs for questionnaire software, and £16,542 in overheads. Based on the average number of people starting at a UK university in 2012 (N = 1,565) [12] the per-person cost to roll out U@Uni to another university was estimated to be £19.16 (the per-person cost to roll out to all UK universities was very similar = £20.64).

### Within-trial cost-effectiveness results (short-term 6-month time horizon)

**Figure 2:**
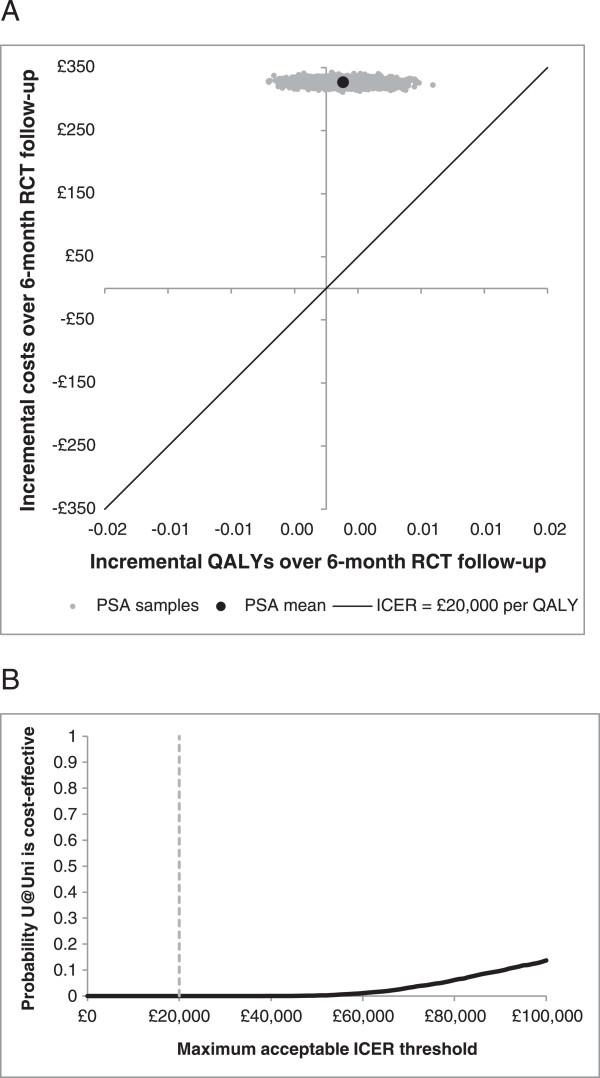
**Within-trial 6-month cost-effectiveness planes and cost-effectiveness acceptability curves for U@Uni compared to do nothing.** Presents the individual-level cost-effectiveness plane and cost-effectiveness acceptability curve resulting from the 5,000 bootstrap replicates in the within-trial cost-effectiveness analysis. **A)** Cost-effectiveness plane showing the per-person incremental 6-month within-trial costs and incremental 6-month within-trial QALYs for full development and implementation of U@Uni compared to do nothing. **B)** Cost-effectiveness acceptability curve showing the probability (out of 5,000 bootstrap replicates) of full development and implementation of U@Uni being cost-effective compared to do nothing at different willingness-to-pay thresholds.

The results of the within-trial cost-effectiveness analysis suggest that over a 6-month time horizon the U@Uni intervention would cost an additional £326 per participant and generate an additional 0.0013 QALYs per person compared to the control condition. The total mean costs were £148.69 in the control arm and £474.96 in the intervention arm. The additional costs in the intervention arm were primarily a result of the intervention cost (£283.29) and slightly more inpatient admissions in the U@Uni arm of the trial than the control arm (an average of 0.06 compared to 0.04 admissions per person over the 6-month RCT follow-up period). The incremental costs and QALYs resulted in an ICER for U@Uni of £243,926 (95% confidence interval £234,805 to £252,873) per additional QALY gained. This is a much higher cost per QALY than bodies such as NICE would be prepared to accept and suggests that in the short term, the U@Uni intervention would not be considered cost-effective. Indeed, the uncertainty analysis found that U@Uni has a 0% probability of being considered cost-effective at the commonly used willingness-to-pay threshold of £20,000 per QALY (see Figure 2).

### Long-term economic modelling results (lifetime time horizon)

Detailed results of the long-term economic modelling are presented in Table 3. The analysis estimates that the changes in health behaviours resulting from U@Uni would produce a small increase in mean survival of approximately 0.0008 years (0.29 days) per person (discounted at 1.5% per annum) compared to the control condition. The estimated gains in health-related quality of life are appreciably larger than the survival benefits, and the modelling suggests that U@Uni would generate an additional 0.0128 discounted QALYs per person compared to the control condition. A reduction in smoking prevalence due to the U@Uni intervention was found to be a statistically significant result in the trial. The model suggests that an individual’s propensity to smoke is 6.3% lower in the intervention arm compared with the control arm.

**Figure 3:**
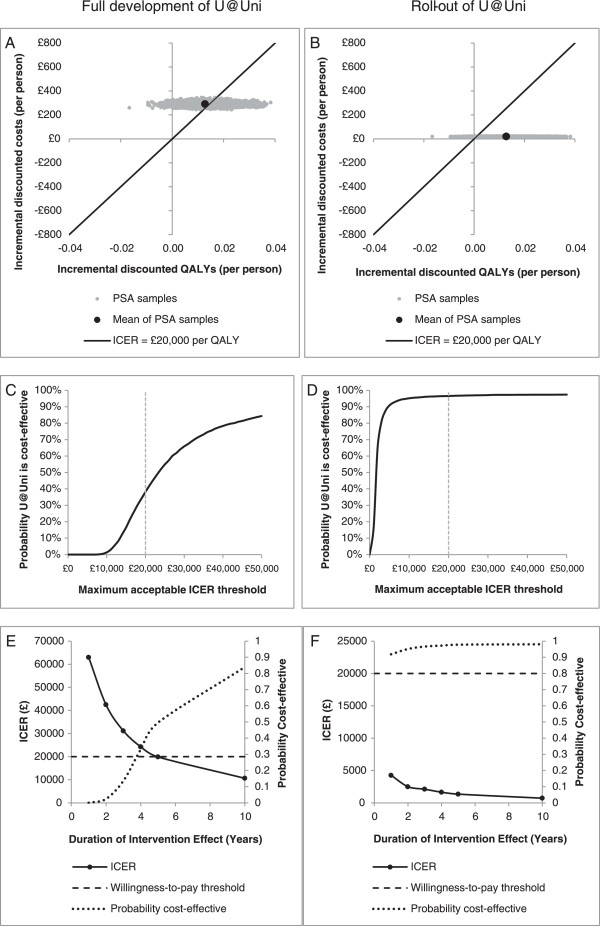
**Long-term cost-effectiveness planes and cost-effectiveness acceptability curves for U@Uni compared to do nothing.** PSA = Probabilistic sensitivity analysis. **A)** Cost-effectiveness plane showing the per-person incremental discounted lifetime costs and incremental discounted lifetime QALYs for full development and implementation of U@Uni compared to do nothing. **B)** Cost-effectiveness plane showing the per-person incremental discounted lifetime costs and incremental discounted lifetime QALYs for roll-out of U@Uni compared to do nothing. **C)** Cost-effectiveness acceptability curve showing the probability (out of 5,000 PSA runs) of full development and implementation of U@Uni being cost-effective compared to do nothing at different willingness-to-pay thresholds. **D)** Cost-effectiveness acceptability curve showing the probability (out of 5,000 PSA runs) of U@Uni roll-out being cost-effective compared to do nothing at different willingness-to-pay thresholds. **E)** A line chart showing the ICER and the probability (out of 5,000 PSA runs) of full development and implementation of U@Uni being cost-effective compared to do nothing at different intervention effect durations. **F)** A line chart showing the ICER and the probability (out of 5,000 PSA runs) of U@Uni roll-out being cost-effective compared to do nothing at different intervention effect durations.

For the full development and implementation of U@Uni as per the University Sheffield RCT, it is estimated that the ICER would be £22,844 per additional QALY gained (jack-knife 95% confidence interval based on 5,000 model runs £22,501 to £23,185) versus control. At the £20,000 per QALY threshold, the full development and implementation of U@Uni would have a 38% probability of being cost-effective compared to the control condition (see Figure 3A and C). The cost per smoker avoided is £55,882.

For the roll-out of U@Uni to other universities, the cost per additional QALY gained is estimated to be £1,545 (jack-knife 95% confidence interval £1,521 to £1,568) versus control. Clearly, this is substantially better than the £20,000 per QALY threshold and would be likely to be considered very cost-effective. The uncertainty analysis estimates a 97% probability that U@Uni roll-out would be cost-effective at the £20,000 per QALY threshold (see Figure 3B and D). The cost per smoker avoided is £3,778.

**Table 3:** **The long-term cost-effectiveness of U@Uni results (all results are per person)**

	Do nothing	U@Uni	Incremental*
**Scenario 1: Full development and implementation of U@Uni**			
Discounted life years	39.5088	39.5096	0.0008
Discounted QALYs	33.2426	33.2553	0.0128
Discounted costs	£0.00	£291.53	£291.53
ICER	-	-	£22,844
Net monetary benefit (NMB)** at threshold of £20,000 per QALY	-	-	-£36.30
Probability U@Uni is cost-effective at willingness-to-pay threshold of £20,000 per QALY	-	-	38.18%
Cost per smoker avoided with U@Uni	-	-	£55,882
**Scenario 2: Roll-out of U@Uni**			
Discounted life years	39.5088	39.5096	0.0008
Discounted QALYs	33.2426	33.2553	0.0128
Discounted costs	£0.00	£19.71	£19.71
ICER	-	-	£1,545
Net monetary benefit (NMB)** at threshold of £20,000 per QALY	-	-	£236
Probability U@Uni is cost-effective at willingness-to-pay threshold of £20,000 per QALY	-	-	96.54%
Cost per smoker avoided with U@Uni	-	-	£3,778
**Threshold cost analysis**			
	**U@Uni price**	**ICER**	**Probability U@Uni is cost-effective at £20 K threshold**
	£0	£0	97.64%
	£5	£392	97.56%
	£10	£784	97.32%
	£25	£1,959	96.26%
	£50	£3,918	94.34%
	£100	£7,836	87.36%
	£150	£11,754	77.54%
	£200	£15,672	64.38%
	£255	£19,981	48.24%

*Any apparent discrepancies are due to rounding.

**Net monetary benefit = incremental QALYs x willingness-to-pay threshold – incremental costs.

The cost threshold analysis suggested that the U@Uni intervention could be priced at anything up to £255 per participant in order to be cost-effective at a £20,000 per QALY threshold (see Table 3). At a price of £160 per person U@Uni would have a 75% probability of being cost-effective and at a price of £85 per person U@Uni would have a 90% probability of being cost-effective. If U@Uni were provided at zero cost there would be a 98% probability that it would be cost-effective (the remaining 2% represents the chance that U@Uni could generate fewer QALYs than the control condition).

Sensitivity analyses suggest that the results are not sensitive to changes in the discount rate used (ICER = £24,324 for full development and implementation and ICER = £1,645 for roll-out) but are very sensitive to the use of utility weights that did not account for the effects of health behaviours on quality of life (ICER = £664,205 for full development and implementation and ICER = £44,916 for roll-out). If the potential quality of life benefits of improving health behaviours are not included in the modelling, then the results suggest that U@Uni would not be considered cost-effective on the basis of estimated survival benefits alone, even at the cheaper roll-out cost. This reiterates the earlier finding that it is the estimated health-related quality of life benefits related to the behaviour changes within the model that are the important drivers when considering cost-effectiveness.

The results are very sensitive to modification of the duration of intervention effect (see Figure 3E and F). The roll-out of U@Uni would have a 92% probability of being cost-effective even if the duration of effect is minimal (one year; ICER = £4,261), whereas the full development of the intervention only becomes likely to be cost-effective if the duration of effect is more than five years. Given the lack of published data on the long-term effect of internet-based interventions, this is an area that would benefit from further research.

### Value of information analysis

**Figure 4:**
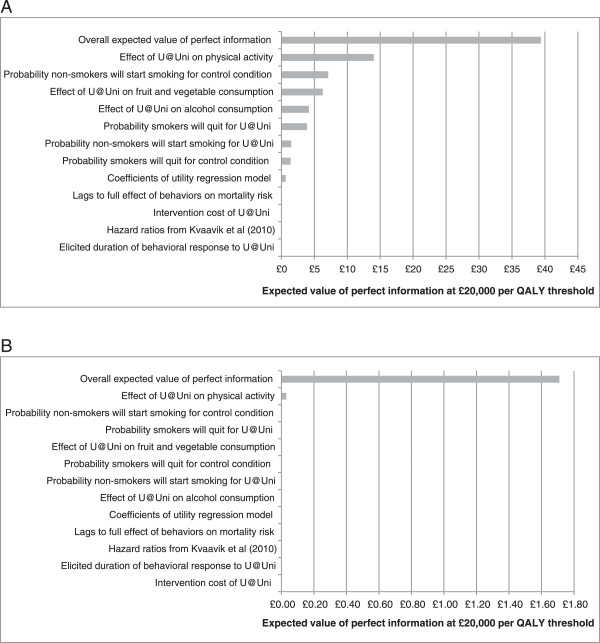
**Expected value of overall and parameter perfect information per person.** Expected value of overall perfect information per person and expected value of perfect information for individual parameters per person at a willingness-to-pay threshold of £20,000 per QALY. **A)** Full development and implementation of U@Uni. **B)** Roll-out of U@Uni to another university.

The results suggest that the overall value of information (as quantified by the overall expected value of perfect information) for the full development and implementation of U@Uni was £39 per person and that the parameters with the highest expected value of information were the coefficients for the effect of U@Uni on health behaviours. Using a target population equal to the number of people starting university in the UK in 2012 (N = 464,891) [12], the overall population value of information was £18.3 million in the first year of the U@Uni intervention. The expected value of information suggests that the cost of the decision uncertainty is £39 per person and therefore society should not be willing to pay more than £39 per person to collect further evidence to resolve the uncertainty in the model outputs. A further interpretation is that the expected value of perfect information is the “opportunity loss” of making the decision about whether to fund U@Uni now rather than waiting for additional evidence to be collected to reduce the decision uncertainty. The two most important parameters driving the decision uncertainty in the model are, firstly, the effectiveness of U@Uni on physical activity, and secondly, the probability that non-smokers will start smoking in the control condition (see Figure 4A).

The results suggest that the overall expected value of perfect information for the roll-out of U@Uni was £1.71 per person and that the most important parameter driving the decision uncertainty in the model was the coefficient for the effect of U@Uni on physical activity (see Figure 4B). Using the same target population as above, the overall population value of information was calculated as £794,964 in the first year of roll-out.

## Discussion

Data from a RCT of a theory-based online health behaviour intervention for new university students (“U@Uni”) were used to estimate the cost of the intervention and its short-term cost-effectiveness. The RCT data were also combined with evidence from the literature and population level datasets in an economic modelling analysis to estimate the long-term cost-effectiveness of the intervention. The costing results suggested that the intervention would cost £208,500 (£283 per student) to fully develop and implement and £29,988 (£19 per student) to roll out to another university. The economic analysis results suggested that U@Uni would not be cost-effective over a 6-month time horizon; this result is not unexpected given that the effects of the intervention on QALYs are likely to be longer term. The lifetime horizon economic analysis suggested that that the full development and implementation of U@Uni had a 38% probability of being cost-effective over a lifetime and that roll-out of U@Uni (i.e., excluding sunk costs) had a 97% probability of being cost-effective over a lifetime. These results were more sensitive to the component of the model estimating the relationship between health behaviours and changes in health-related quality of life than the components relating to mortality risk.

The long-term modelling indicates that a QALY gain of 0.0128 per person is obtained from the intervention. This is a relatively small number and it might be expected that the cost-effectiveness of the intervention would be highly sensitive to costs. However, the value of information analysis did not find the cost data to be an important driver of decision uncertainty in the model (Figure 4). Furthermore, the cost-threshold analysis indicates that the U@Uni roll-out could be priced at anything up to £255 per person and still be likely to be cost-effective. Given that the actual roll-out costs are under £20 per person, prices would therefore have to increase by over 10 fold to approach the threshold. This indicates that in fact the ICER is not particularly sensitive to costs, despite the small QALY value.

The cost-effectiveness of roll-out of U@Uni compares favourably with other public health and health behaviour interventions and the full development and implementation of U@Uni is not excessively high compared to previously published evidence on the cost-effectiveness of similar behavioural interventions. In 2007, Gordon et al. conducted a review of cost-effectiveness studies of face-to-face behavioural interventions for smoking, physical activity, diet and alcohol [24]. The review found that ICERs for most face-to-face multiple behaviour interventions ranged from cost saving to €24,691 (2006 Euros; approximately £24,187 at 2011/2012 prices) per QALY. The ICER for full development and implementation of U@Uni (£22,844) falls towards the upper end of this range and the ICER for roll-out of U@Uni falls towards the lower end of the range, suggesting that an online multiple behaviour intervention can generate a similar ratio of costs to benefits as that generated by face-to-face interventions. In 2009, Tate et al. conducted a review of the cost-effectiveness of internet interventions [25]. They found a lack of published economic evidence relating to internet-based interventions, and none of the studies they identified were targeted at improving general population health behaviours. Although this has improved somewhat in recent years, there remains a paucity of published economic evaluations of internet based interventions to improve multiple health behaviours. To our knowledge, the current study is the first to analyse the cost-effectiveness of such an intervention in university students, and so should be a useful addition to the growing body of literature on internet-based interventions.

The current study provides estimates of both the sunk costs and roll-out costs of an internet intervention targeted at multiple health behaviour change. In 2012, Owen et al. published a review of the cost-effectiveness of public health interventions in the UK based on evidence from evaluations conducted by NICE [26]. The review found that the majority (71%) of ICERs for public health interventions were below £20,000 per QALY, as was the case for the roll-out cost of U@Uni in the current study. Of the 200 cost-effectiveness estimates reviewed by Owen et al., seven cost between £20,000 and £30,000 per QALY as was the case for the full development and implementation of U@Uni in the current study.

The methods used in the current study have some limitations. First, the economic modelling took a broad approach, using risk of mortality from an epidemiological study by Kvaavik et al. [11] to translate behaviour change into long-term QALYs. An alternative approach would be to incorporate the effects of health behaviours on risk of individual diseases such as diabetes, coronary heart disease and cancers, and model mortality and quality of life as a function of these diseases rather than directly as a function of health behaviours. This additional level of detail could have generated different estimates of the cost-effectiveness of U@Uni. Second, because morbidity was not explicitly modelled, the economic analysis only included the cost of the U@Uni intervention and did not take account of ongoing long-term costs of morbidity, e.g., the cost to the NHS of caring for diabetes, heart attacks and cancers. The authors consider this a conservative structural assumption because improvements in health behaviours as a result of U@Uni would be expected to produce savings in health care costs over the longer-term. A third limitation is that the economic model only partially accounts for the correlation between health behaviours at the individual level. Correlations at baseline are incorporated from the U@Uni RCT data; however, in the long-term extrapolation, the model assumes individuals maintained their behaviours exactly as per their percentile ranks in the Health Survey for England 2008 general population data [1], and correlations in the change in behaviours over six months are not incorporated as each health behaviour change is simulated independently. A fourth limitation is the lack of published data on duration of intervention effect, meaning that expert elicitation had to be sought to populate this parameter. The sensitivity analysis results indicate that roll-out of the intervention is still highly likely to be cost-effective even if the effect endures only one year. However, cost-effectiveness of the full design and implementation of the intervention is highly sensitive to duration of effect. A final limitation is that the cost analysis was primarily based on retrospectively recalled self-report of the number of hours spent developing and implementing U@Uni, whereas a prospective micro-costing study may have provided a more accurate estimate of the cost of the intervention.

The expected value of information analysis suggested that the effect of U@Uni on fruit and vegetable consumption, physical activity, alcohol consumption and smoking were the model parameters most worth investing in as part of any future research. Reducing the uncertainty in these parameters could substantially reduce the decision uncertainty about whether to fund U@Uni. The U@Uni RCT included some methodological challenges [7], and conducting an additional RCT to overcome these challenges and better estimate the effect of U@Uni on health behaviours could reduce the model and decision uncertainty. For the decision about whether to roll out U@Uni to other universities, the value of information analysis suggested that provided the cost of obtaining evidence from an additional RCT was well below the estimated £794,964 threshold then the research would be considered worthwhile. Other further research could focus on taking a much more detailed and expanded economic modelling approach, e.g., explicitly simulating significant morbidities such as diabetes, heart disease and cancers, and using alternative methods for modelling the trajectories of behaviours over time and the interactions between health behaviours. Finally, the use of the model framework for evaluation of other interventions targeted at single or multiple health behaviours could be explored. If population characteristics, the cost of the intervention, evidence of the effect of the intervention on health behaviour(s), and assumptions regarding the duration of effect were available the model could be very easily adapted in this way.

In the UK, NICE have the remit of evaluating health technologies and making recommendations to the Department of Health about whether they should be funded or not. Based on the economic analysis in this study the implication is that, given a NICE willingness-to-pay threshold of £20,000 per QALY [8], the implementation of U@Uni could be recommended as cost-effective provided the roll-out costs were low enough per participant (i.e., more of the order of the £19 per participant estimated here and not as high as the total up-front costs of £283 per participant for the full development of U@Uni at the University of Sheffield). It has been assumed that the health behaviour effects observed in the U@Uni RCT can be generalised to other universities across the UK. It is unclear at this stage whether the results can be generalised internationally or to subgroups of the general population other than new university students, but any online health behaviour intervention that could generate similar changes in health behaviour for similar costs could potentially be expected to have a similar level of cost-effectiveness.

## Conclusions

This study provides the first estimate of the full implementation (£208,500) and roll-out (£29,988) costs of an online intervention targeting multiple health behaviours in new university students. The study is also the first to explore the cost-effectiveness of an online multiple health behaviour intervention, providing estimates of both the short-term (£243,926) and long-term (£22,844 for full implementation and £1,545 for roll-out) cost per QALY of U@Uni. The results suggest that based upon the assumptions of the long-term modelling, the roll-out of U@Uni to other universities would most likely be a cost-effective option for the UK Department of Health, whereas the full implementation would not. Uncertainty analysis suggests that further research (e.g., via a second RCT) that could reduce uncertainty in the effectiveness of U@Uni on health behaviours, particularly around changes in physical activity and duration of intervention effect, would be potentially valuable to decision makers. The new economic modelling framework developed for this analysis enables estimation of the effects of changes in fruit and vegetable consumption, physical activity, alcohol consumption and smoking into long-term costs and QALYs, and as such could be used to evaluate a broad range of public health interventions.

## Electronic supplementary material

Additional file 1: **Detailed methods and results for economic evaluation.** (DOCX 56 KB)

## Abbreviations

ICER: Incremental cost-effectiveness ratio
QALY: Quality-adjusted life year
RCT: Randomised controlled trial
UK: United Kingdom.

## References

[1] National Centre for Social Research (2008). Health Survey for England 2008.

[2] Parliamentary Office of Science and Technology (2007). Postnote number 283: Health Behaviour.

[3] Epton T, Norman P, Sheeran P, Harris PR, Webb TL, Ciravegna F, Brennan A, Meier P, Julious SA, Naughton D, Petroczi A, Dadzie A, Kruger J (2013). A theory-based online health behavior intervention for new university students: study protocol. BMC Public Health.

[4] Harris PR, Epton T (2009). The impact of self-affirmation on health cognition, health behavior and other health-related responses: a narrative review. Soc Pers Psychol Comp.

[5] Ajzen I (1988). Attitudes, Personality and Behaviour.

[6] Gollwitzer PM, Sheeran P (2006). Implementation intentions and goal achievement: a meta-analysis of effects and processes. Adv Exp Soc Psychol.

[7] Epton T, Norman P, Dadzie AS, Harris PR, Webb TL, Sheeran P, Julious SA, Ciravegna F, Brennan A, Meier PS, Naughton D, Petroxzi A, Kruger J, Shah I (2014). A theory-based online health behaviour intervention for new university students (U@Uni): results from a randomised controlled trial. BMC Public Health.

[8] National Institute for Health and Care Excellence (2012). Methods for the development of NICE public health guidance (3rd edition).

[9] Drummond MF, Sculpher MJ, Torrance GW, O'Brien BJ, Stoddart GL (2005). Methods for the Economic Evaluation of Health Care Programmes.

[10] Dolan P, Gudex C, Kind P, Williams A (1995). A social tariff for EuroQoL: Results from a UK general population survey. Discussion Paper No. 138. Centre for Health Economics.

[11] Kvaavik E, Batty D, Ursin G, Huxley R, Gale CR (2010). Influence of individual and combined health behaviours on total and cause-specific mortality in men and women. Arch Intern Med.

[12] Universities and Colleges Admissions Service (UCAS): **Institution level statistics 2009–2013.** Available from http://www.ucas.com/data-analysis/data-resources/data-tables/acceptances-institution-domicile-group-and-entry-year (same data on former website accessed 29 May 2013)

[13] Department of Health (2012). NHS reference costs: financial year 2011 to 2012.

[14] Curtis L, Curtis CL (2012). Unit Costs of Health and Social Care 2012. Canterbury.

[15] Brazier J, Ratcliffe J, Salomon JA, Tsuchiya A (2007). Measuring and Valuing Health Benefits for Economic Evaluation.

[16] Manca A, Hawkins N, Sculpher MJ (2005). Estimating mean QALYs in trial-based cost-effectiveness analysis: the importance of controlling for baseline utility. Health Econ.

[17] Office for National Statistics (2013). England and Wales Interim Life Tables 2009-11.

[18] van Hout BA, Al MJ, Gordon GS, Rutten FFH (1994). Costs, effects and C/E-ratios alongside a clinical trial. Health Econ.

[19] Brennan A, Kharroubi S, O'Hagan A, Chilcott J (2007). Calculating partial expected value of perfect information via Monte Carlo Sampling Algorithms. Med Decis Making.

[20] Sculpher MJ, Claxton K (2005). Establishing the cost-effectiveness of new pharmaceuticals under conditions of uncertainty - when is there sufficient evidence?. Value Health.

[21] Strong M, Oakley JE (2012). An Efficient Method for Computing Single-Parameter Partial Expected Value of Perfect Information. Med Decis Making.

[22] National Institute for Health and Care Excellence: **Guide to the methods of technology appraisal. 2013, section 5.6.** Available from http://www.nice.org.uk/article/PMG9 (accessed 29 May 2013) 27905712

[23] Ara R, Brazier J (2011). Populating an economic model with health state utility values: moving towards better practice. Discussion Paper No. 09/11. Health Economics and Decision Science.

[24] Gordon L, Graves N, Hawkes A, Eakin E (2007). A review of the cost-effectiveness of face-to-face behavioural interventions for smoking, physical activity, diet and alcohol. Chronic Illness.

[25] Tate DF, Finkelstein EA, Khavjou O, Gustafson A (2009). Cost effectiveness of internet interventions: review and recommendations. Ann Behav Med.

[26] Owen L, Morgan A, Fischer A, Ellis S, Hoy A, Kelly MP (2012). The cost-effectiveness of public health interventions. J Public Health.

[CR27] The pre-publication history for this paper can be accessed here:http://www.biomedcentral.com/1471-2458/14/1011/prepub

