# Supplementary material for: The cost-effectiveness of a theory-based online health behaviour intervention for new university students: an economic evaluation
Source: BMC Public Health. 2014 Sep 27;14:1011. doi: 10.1186/1471-2458-14-1011 (PMC4195974; doi:10.1186/1471-2458-14-1011)
Supplement: Supplementary file 1 — Additional file 1: Detailed methods and results for economic evaluation. (DOCX 56 KB) [file 12889_2014_7137_MOESM1_ESM.docx]

**Additional file 1: Detailed methods and results for economic evaluation**

**A. Details of costing analysis methods**

The cost of U@Uni was estimated in two primary ways: first, by estimating the full cost of developing and implementing the U@Uni intervention from scratch (i.e., the full cost as implemented in the University of Sheffield) and, second, by estimating the cost of rolling out U@Uni to another university (i.e., without the sunk costs of development).

*Data collection*

To estimate the full cost, a survey was designed to collect data from the staff involved in developing and implementing the intervention (three Professors of Psychology, one Senior Lecturer in Psychology, one Research Associate in Psychology, one Research Associate in Computer Science and one Temporary Casual Worker in Computer Science). The survey asked respondents to estimate the number of hours (or days, if preferred by the respondent) that they had spent on different aspects of the development and implementation of the intervention. Separate surveys were sent to Department of Psychology and Department of Computer Science staff. The lead researcher from each department was also asked to estimate non-staff costs attributable to the development and implementation of U@Uni. All surveys also asked for the respondent’s job title, pay grade, the average number of hours per day they spend at work and the average number of days per week they spend at work. The surveys included the following instructions:

- Please only include time spent on DEVELOPMENT of the intervention, NOT time spent on RESEARCH or EVALUATION of the intervention
- We are trying to estimate the amount the intervention costs to develop and deliver, not the cost of the research project
- Please DO include ALL TIME spent doing each activity, not just time since the project officially launched
- Please DO include time spent working INDIVIDUALLY (e.g., at the office, working at home, out around Sheffield)
- Please DO include time spent working WITH COLLEAGUES (e.g., meetings, discussions, collaborative activities)
- Please DO NOT include time spent in meetings that were related only to the research project (e.g., bid preparation meetings, dependent variable development meetings)
- Please DO NOT include time spent purely for research (e.g., developing dependent variables, working with Survey Gizmo, analysing trial data)
- Where possible please enter the number of hours or days that you have actually worked (i.e., up to the end of the trial)
- Where it is not possible to enter the number of hours or days that you actually work please enter an estimate of the future workload (e.g., updating the intervention)

The surveys for Psychologists had the following aspects of development and implementation:

Table A1. Department of Psychology cost aspects included in full cost of U@Uni

| **Staff aspects** | **Non-staff aspects** |
| --- | --- |
| - Elicitation studies for initial formative research (to elicit important health beliefs from students) - Questionnaire studies for initial formative research - Developing health messages - Developing self-affirmation task - Developing implementation intentions module - Development of local elements - Design (communication with graphic designers) - Promotion - Monitor facebook group - Moderate other user groups - Monitor intervention use - Monitoring website functionality - Communication with users - Other monitoring - Updating local elements - Updating health messages - Updating self-affirmation task - Updating implementation intentions module - Designing the website version - Designing the mobile phone version | - Prize money for participation in initial formative research (pilot) - Payments for health messages for participation in interviews and focus groups to determine health messages - Payments for test users for testing the intervention - Advertising - Printing - Survey software |

The surveys for Computer Scientists had the following aspects of development and implementation:

Table A2. Department of Computer Science cost aspects included in full cost of U@Uni

| **Staff aspects** | **Non-staff aspects** |
| --- | --- |
| - Developing the website version (desktop) - Developing the website version (mobile) - Developing the mobile phone version (Android) - Developing the mobile phone version (iOS) - Setting up the log analysis and building the project admin console - Front end design - Designing and developing facebook page & twitter account interaction - Designing and developing Google+ page - Website monitoring and maintenance (desktop and mobile) - Mobile phone version monitoring and maintenance (Android and iOS) - Monitoring intervention usage (website and mobile phone versions) - Communication with users - Monitoring and maintenance of database / backend - Updating the website version (desktop and mobile) - Updating the mobile phone version (Android and iOS) - Updating the tablet version - Designing the website version (desktop and mobile) - Designing the mobile phone version (Android and iOS) - Back-end design (across all deployment options) | - Hardware - Server space: testing - Server space: long-term - Domain name - Charges for The Cloud - Storage of intervention content data - Storage of usage data - Storage of other data - Charges for communications |

The lead Computer Science researcher reported that the university’s existing hardware, software, sever space and domain names were used to develop the intervention. It was assumed in all analyses that other universities would also be able to use existing computer equipment and therefore there were no non-staff computer science costs.

*Estimating the full cost of development and implementation*

The total hours of time each member of staff spent on development and implementation of U@Uni were calculated from their survey responses. Uncertainty around the estimates was captured by asking survey respondents to give upper and lower as well as central estimates of the amount of time they spent developing and implementing U@Uni. Three respondents did this. One respondent reported they had provided the actual amount of time spent rather than an estimate therefore their hours were treated as certain. For the remaining respondents, the lower estimate was assumed to be 89% of their central estimate and the upper estimate was assumed to be 125% of their central estimate, both based on the average of the ratios of upper and lower to central estimates from the three respondents providing this information.

The numbers of hours for each staff member were input into the University Research Management System (URMS) to calculate the total cost of staff time (including salary, pension contributions and national insurance), the cost of overheads and indirect costs associated with staff time (calculated automatically by URMS) and the cost of non-staff resources such as questionnaire software and payments to participants in formative research. It was assumed to take 12 months to fully develop and implement the U@Uni intervention.

The average cost of the intervention was estimated by summing all staff members’ central estimates of costs (including overheads and indirect costs) and the non-staff costs. 95% confidence intervals were estimated by summing the lower and upper estimates of the staff members’ costs (including overheads and indirect costs) and the non-staff costs.

The health economic researchers considered there to be additional parameter uncertainty because the actual amount of time spent on developing the intervention, the staff salaries, and the non-staff resource costs may all have been different under different circumstances (for example if the intervention had been implemented at a university other than the University of Sheffield). The upper and lower limits were widened by a further 50% to reflect this additional uncertainty, i.e.,

Final upper limit = initial upper limit + (initial upper limit – central estimate) * 1.5

Final lower limit = initial lower limit – (central estimate - initial lower limit) * 1.5

This analysis resulted in a mean cost of £208,500 (95% confidence interval £194,723 to £238,611).

These results were divided by the number of people receiving the intervention in the U@Uni RCT (N = 736) [1] to give a per-person cost of £283.29 (95% CI £265 to £324). These parameters were used to define a distribution for costs in R and the results were a lognormal distribution with parameters on the normal scale of mean = 12.276 and standard deviation = 0.051. This distribution was used to define the cost parameter for the PSA in the simulation model.

*Estimating the roll-out cost of implementation*

To approximate the cost of rolling out the intervention to another university, a subset of data from the full costing analysis was used. The following aspects of development and implementation were included:

Table A3. Cost aspects included in roll-out cost of U@Uni

| **Staff aspects** | **Non-staff aspects** |
| --- | --- |
| - Development of local elements (Psychology) - Promotion (Psychology) - Monitor facebook group (Psychology) - Moderate other user groups (Psychology) - Monitor intervention use (Psychology) - Monitoring website functionality (Psychology) - Communication with users (Psychology) - Other monitoring (Psychology) - Designing and developing facebook page & twitter account interaction (Computer Science) - Designing and developing Google+ page (Computer Science) - Website monitoring and maintenance (desktop and mobile) (Computer Science) - Mobile phone version monitoring and maintenance (Android and iOS) (Computer Science) - Monitoring intervention usage (website and mobile phone versions) (Computer Science) - Communication with users (Computer Science) - Monitoring and maintaining database/backend (Computer Science) | - Payments for test users (Psychology) |

None of the survey respondents reported any time spent on updating the intervention therefore this was not included in the estimation of roll-out costs.

Overheads, indirect costs and 95% confidence intervals were calculated as described above for the full cost analysis using URMS. It was assumed to take 6 months to roll-out the U@Uni intervention. This analysis resulted in a mean cost of £29,988 (95% confidence interval £27,054 to £35,081). These were divided by the average number of people starting university at universities in the UK in 2012 (N = 1,565) [2] to give a per-person cost of £19.16 (95% CI £17 to £22). These parameters were used to define a distribution for costs in R and the results were a lognormal distribution with parameters mean = 10.335 standard deviation = 0.066.

The 95% confidence intervals were widened by an additional 50% at the upper and lower bounds to reflect additional uncertainty about the amount of resources and unit costs of staff time that would be required to implement the U@Uni intervention. This method was used to account for the fact that estimates of staff time were collected retrospectively and therefore may have included inaccuracies, and that the cost of fully implementing U@Uni may have been slightly different if other staff had undertaken the development work. Probability distributions were fitted to the central estimates and widened confidence intervals using R software [3]. These distributions were then used as parameter inputs for the economic model.

The researchers considered another analysis to estimate the cost of full national roll-out, by multiplying the roll-out costs by the number of universities in the UK in 2012 (N = 320) [2] and dividing by the total number of people starting university at universities in the UK in 2012 (N = 464,910) [2], but this gave an extremely close value to that above (mean = £20) therefore only one roll-out economic evaluation was conducted.

**B. Unit costs used in within-trial cost-effectiveness analysis**

Table B1. Unit costs

| **Resource** | **Unit cost** | **Notes** | **Source** |
| --- | --- | --- | --- |
| GP visit | £36 | 11.7 min surgery consultation excluding qualifications, including direct care staff costs) | Table 10.8b of PSSRU Unit Costs 2012 [4] |
| Hospital inpatient admission | £1,713 | Weighted average of elective and long- and short-stay non-elective admissions from 'Index' sheet | EI, NEI_S and NEI_L NHS Reference Costs 2011-12 NSRC01 [5] |
| Hospital outpatient attendance | £105.89 | Total from 'OPATT' sheet | NHS Reference Costs 2011-12 NSRC01 [5] |
| Emergency department attendance | £121.93 | Weighted average from 'Index' sheet | AandEMSAD and AandEMSNA NHS Reference Costs 2011-12 NSRC01 [5] |
| Ambulance services | £214.02 | Total from 'Index' sheet | AMB_SEE NHS Reference Costs 2011-12 NSRC01 [5] |

**C. Details of treatment effectiveness regression methods used for economic modelling**

The baseline (pre-U@Uni) behaviours for each simulated individual were input into the economic model based on baseline data from the U@Uni RCT [1]. The model predicted the year 1 continuous behaviours (i.e., fruit and veg, alcohol, physical activity) under the control condition and under the intervention condition using ordinary least squares linear regression models of the RCT data [1] The simulation model assumed the 6-month observed behaviour in the RCT remains constant to 12-months. The results of the linear models are outlined below.

Table C1. Results of treatment effectiveness regression models used in economic model

| Predictor variable | Fruit and vegetable consumption at 12 months (portions per day) | | Alcohol consumption at 12 months (units per week) | | Physical activity at 12 months (minutes per week) | |
| --- | --- | --- | --- | --- | --- | --- |
|  | Coefficient | Standard error | Coefficient | Standard error | Coefficient | Standard error |
| Constant | 3.010 | 1.146 | 18.922 | 4.583 | 161.333 | 28.946 |
| Baseline behaviour | 0.248 | 0.031 | 0.483 | 0.032 | 0.234 | 0.030 |
| Age | 0.059 | 0.058 | -0.577 | 0.232 | -2.776 | 1.478 |
| Gender (1 = male; 0 = female) | -0.025 | 0.323 | -0.425 | 1.268 | -2.019 | 7.730 |
| Intervention (1= U@Uni; 0 = control) | -0.112 | 0.311 | -0.428 | 4.583 | 4.014 | 7.340 |
| Adjusted R^2^ | 0.059 | | 0.178 | | 0.067 | |

As an example, for fruit and vegetable consumption the equation for predicted 12-month fruit and vegetable consumption was:

FV_1_ = 3.010 + 0.248(FV_0_) + 0.059(age) - 0.025(gender) - 0.112(intervention)

Where

FV_1_ = fruit and vegetable portions per day at 12 months

FV_0_ = fruit and vegetable portions per day at baseline

age = age in years

gender = 1 if male and = 0 if female

intervention = 1 if received U@Uni and = 0 if received control

Table C2. Covariance matrices for treatment effectiveness regression models

| **Fruit and vegetables (portions per day)** | | | | | |
| --- | --- | --- | --- | --- | --- |
|  | Intervention | Baseline fruit and vegetables | Age | Gender | Constant |
| Intervention | 0.096466 |  |  |  |  |
| Baseline fruit and vegetables | -0.000037 | 0.000937 |  |  |  |
| Age | 0.001252 | 0.000010 | 0.003308 |  |  |
| Gender | 0.005955 | -0.000730 | 0.000559 | 0.104122 |  |
| Constant | -0.073004 | -0.005975 | -0.063405 | -0.046807 | 1.313950 |
| **Alcohol (units per week)** | | | | | |
|  | Intervention | Baseline alcohol | Age | Gender | Constant |
| Intervention | 1.490600 |  |  |  |  |
| Baseline alcohol | -0.000159 | 0.001041 |  |  |  |
| Age | 0.019172 | 0.000445 | 0.053870 |  |  |
| Gender | 0.094133 | -0.002663 | 0.008914 | 1.606729 |  |
| Constant | -1.133062 | -0.019606 | -1.035419 | -0.771976 | 20.999992 |
| **Physical activity (minutes per week)** | | | | | |
|  | Intervention | Baseline physical activity | Age | Gender | Constant |
| Intervention | 53.873807 |  |  |  |  |
| Baseline physical activity | 0.000259 | 0.000883 |  |  |  |
| Age | 1.132597 | -0.002279 | 2.183811 |  |  |
| Gender | 3.921136 | -0.021960 | 0.260070 | 59.758110 |  |
| Constant | -48.937143 | -0.088131 | -41.383532 | -23.773473 | 837.873760 |

The linear prediction from the ordinary least squares regression is an average estimate of the effect of predictor variables on the 12-month behaviours. To simulate the individual variability in response, a random sample from the normal distribution of residuals from each linear regression model was added to the predicted value in the simulation model. The distributions of residuals were as follows:

Table C2. Distributions of individual-level residuals for each treatment effectiveness regression model

|  | Mean | Standard deviation |
| --- | --- | --- |
| Fruit and vegetable consumption model | -3.18E-09 | 4.897 |
| Alcohol consumption model | 1.85E-08 | 19.905 |
| Physical activity | -1.38E-07 | 106.001 |

The predicted continuous behaviours were lower-bounded at 0 in the simulation model so that if the predicted value was less than 0 it was replaced with 0.

Individuals’ 1-year behaviours were simulated using the above regression models under both the control condition and the intervention condition.

For smoking, the model used a beta distributions to estimate the 1-year probabilities of quitting smoking since baseline (for baseline smokers) and of taking up smoking since baseline (for baseline non-smokers). The probabilities were different under the control and intervention conditions:

Table C3. Smoking results from U@Uni RCT [1] used in the economic model

|  | Distribution | α | β | Mean probability |
| --- | --- | --- | --- | --- |
| Probability of smokers quitting - Intervention | Beta | 27 | 33 | 0.450 |
| Probability of non-smokers starting - Intervention | Beta | 14 | 466 | 0.029 |
| Probability of smokers quitting - Control | Beta | 19 | 45 | 0.297 |
| Probability of non-smokers starting - Control | Beta | 27 | 462 | 0.055 |

A random number was compared to the probability of quitting/starting smoking for each individual and if the random number was lower than the event probability then the simulated individual was assumed to have changed their behaviour at year 1.

Trajectories of smoking behaviour were not modelled, i.e., 1-year smoking behaviour was maintained throughout the lifetime.

**D. Details of expert elicitation methods**

*General methods*

Two elicitation exercises were undertaken. The elicitation process for both exercises was based on recommendations made by Oakley [6] and O’Hagan et al [7], for example:

- Experts were given an introduction to the elicitation process including discussion of the undesirability of the alternatives to expert elicitation, discussion of the issue of elicitation versus collecting more data, and clarifying that the researchers were not trying to obtain an artificially precise estimate of the parameter.
- The facilitating researcher gave feedback on each fitted distribution to the experts, highlighting features such as median and percentile values to ascertain whether the fitted distribution was an acceptable representation of the experts’ beliefs.

*Lags to full effect of changing behaviour on mortality*

Two experts were included in the elicitation process: one Professor of Public Health and one Professor of Health Economics and Decision Modelling. One health economic modeller acted as the facilitator.

Experts were asked to express their beliefs about the number of years it would take for a change in behaviour to have its full effect on mortality risk, i.e., at what point previous behaviour would become irrelevant and the person would have the risk they would have had if they had always behaved in the new way.

Behavioural aggregation [7] was used to combine the two experts’ beliefs by conducting group elicitation with both experts at the same time. The elicitation session followed the following process for each behaviour:

1. Introduction to the elicitation process by the facilitator
2. Explanation of the parameter of interest by the facilitator (years until full effect of behaviour change on mortality risk)
3. Discussion between experts
4. Online “trial roulette” exercise using the MATCH elicitation tool software [8]
5. Fitting of parametric distribution using MATCH [8]
6. Feedback to experts on features of fitted parametric distribution
7. Either conclusion of elicitation, or revisiting MATCH [8] task if experts did not consider the parametric distribution an acceptable representation of their joint beliefs

The trial roulette method of elicitation was proposed by Gore in 1987 [9] and is described in Oakley (2010) [6]. In summary, the method requires experts to place “chips” onto a grid to build a histogram to represent their uncertainty about the mean value of a parameter. The exercise used 30 bins representing 1-year each with a range from 0 to 30 years, and the grid was 30 chip spaces high. Experts were initially asked to place 50 chips to represent their uncertainty. Feedback was provided to experts on the implied median of the best-fitting distribution as recommended by the “Fitting and Feedback” tool in the MATCH software [8].

*Duration of behavioural effects of the U@Uni intervention*

Two experts were included in the elicitation process: one Professor of Psychology and one Social Psychology Research Assistant. One health economic modeller acted as the facilitator.

Experts were asked to express their beliefs about the number of years post 6-month behaviour change it would take for individuals’ behaviour to revert to what it would have been had they not received U@Uni: “duration of treatment effect”.

Behavioural aggregation [7] was used to combine the two experts’ beliefs by conducting group elicitation with both experts at the same time. The elicitation session involved two stages:

1. Elicitation of a distribution to represent experts’ beliefs about the individual person-level distribution of duration treatment effect.
2. Elicitation of experts’ uncertainty about the parameters of the individual person-level distribution.

The first stage involved followed these steps:

1. Introduction to the elicitation process by the facilitator
2. Explanation of the question by the facilitator: how many people would have a “duration of treatment effect” lasting x years, lasting x+1 years and so on.
3. Discussion between experts
4. Online “trial roulette” exercise using the MATCH elicitation tool software [8]
5. Fitting of parametric distribution using MATCH [8]
6. Feedback to experts on features of fitted parametric distribution
7. Either conclusion of elicitation, or revisiting MATCH [8] task if experts did not consider the parametric distribution an acceptable representation of their joint beliefs

The trial roulette method of elicitation was proposed by Gore in 1987 [9] and is described in Oakley (2010) [6]. In summary, the method requires experts to place “chips” onto a grid to build a histogram to represent their uncertainty about the mean value of a parameter. The exercise used 30 bins representing 1-year each with a range from 0 to 30 years, and the grid was 30 chip spaces high. Experts were initially asked to place 50 chips to represent their uncertainty. Feedback was provided to experts on the implied median of the best-fitting distribution as recommended by the “Fitting and Feedback” tool in the MATCH software.

Once a parametric distribution was agreed on in stage 1, the facilitator asked the experts to move away from thinking about individual-level variation in duration of treatment effect and think instead about their uncertainty in the mean number of years of treatment effect. The facilitator reported the implied mean of the parametric distribution from stage 1 to the experts and asked them to estimate their upper and lower 95% confidence intervals for the mean value. This task was then repeated for the interquartile range (IQR); the facilitator reported the implied IQR from stage 1 to the experts and asked them to estimate their upper and lower 95% confidence intervals for the range (i.e., how wide could it be and how narrow could it be?).

**E. Details of methods for modelling the effects of health behaviours on mortality risk**

Evidence from a published survival analysis [10] was used to define risk functions for mortality in the economic model. The effects of fruit and vegetable consumption, alcohol consumption, physical activity and smoking on mortality were based on hazard ratios from Cox regressions reported in Table 2 of Kvaavik et al. (2010) [10]:

Table E1. Hazard ratios from Table 2 of Kvaavik et al (2010) [10]

|  | Mean hazard ratio | Lower 95% CI | Upper 95% CI |
| --- | --- | --- | --- |
| Fruit and vegetable (portions per day) (from Table 2) | | | |
| <3 times/day versus >= 3 times/day | 1.10 | 0.96 | 1.25 |
| Alcohol (units per week) (from Table 2) | | | |
| >14/21 (female/male) versus <=14/21 | 1.18 | 1.00 | 1.39 |
| Physical activity (minutes per week) (from Table 2) | | | |
| <2hours/week versus >= 2hours/week | 1.43 | 1.20 | 1.69 |
| Smoking status |  |  |  |
| Smoker versus non-smoker | 1.43 | 1.26 | 1.62 |

Continuous risk functions were developed from the data outlined above by combining the reported hazard ratios with data from the Health Survey for England (HSE) 2008 [11]. The hazard ratios for each category of behaviour were weighted by the number of people from the HSE model data falling into that category to generate an “implied hazard ratio” (where the reference point with a hazard ratio of 1 is the HSE model data mean rather than the reference category from the Kvaavik et al [10] paper). These implied hazard ratios were then plotted against the mean observed behaviour from the HSE data for each behaviour category.

For example, for fruit and vegetable consumption, the implied hazard ratios (HRs) were calculated as follows:

Implied HR for <3 portions per day = HR for <3 portions per day / ((number of individuals in HSE data consuming <3 portions per day * HR for <3 portions per day) + (number of individuals in HSE data consuming >=3 portions per day * HR for >=3 portions per day)) / total n in HSE data

= 1.10 / (((6,006 * 1.10) + (8,919 * 1.00)) / 14,925)

= 1.06

Implied HR for >=3 portions per day = HR for >=3 portions per day / ((number of individuals in HSE data consuming <3 portions per day * HR for <3 portions per day) + (number of individuals in HSE data consuming >=3 portions per day * HR for >=3 portions per day)) / total n in HSE data

= 1.00 / (((6,006 * 1.10) + (8,919 * 1.00)) / 14,925)

= 0.96

The mean portions of fruit and vegetables consumed by individuals in the HSE model data falling in the <3 portions per day and >=3 portions per day categories were 1.46 and 5.20, respectively. These were plotted against the implied HRs:

Figure E1. Implied hazard ratios for fruit and vegetable consumptions’ effect on mortality risk plotted against mean fruit and vegetable consumption from HSE

A logarithmic trendline was fitted to these data points and the slope (-0.076) and constant (1.086) of this line were estimated using the =LINEST() function in Microsoft Excel 2010 and these values were used to define the continuous mortality risk function for portions of fruit and vegetables consumed per day.

Figure E2. Continuous logarithmic risk function for the effect of fruit and vegetable consumption on mortality risk

A continuous log risk function for physical activity (minutes per week) and continuous linear risk functions for men and women’s alcohol consumption (units per week) were estimated in the same way as outlined above for fruit and vegetable portions per day. For alcohol consumption, two separate risk functions were developed for men and women, to account for the fact that the HR reported in Kvaavik et al. [10] referred to a reference category of <14 units per week for women and <21 units per week for men.

Based on examination of the plots, the hazard ratios for 0 minutes physical activity and 0 portions of fruit and vegetables were both assumed to be 1.6.

As smoking status is a categorical behavioural variable, no attempt was made to estimate a continuous risk function for this behaviour. The HRs from Kvaavik et al. (1.43 for smoker and 1.00 for non-smoker) were weighted by prevalence in the HSE model data to estimate implied HRs as outlined above for the other behaviours. The implied HRs for each behaviour are outlined below:

Table E2. Implied hazard ratios for effect of health behaviours on mortality

|  | Implied hazard ratio |
| --- | --- |
| Fruit and vegetable (portions per day) | |
| <3 | 1.06 |
| >=3 | 0.96 |
| Alcohol (units per week) | |
| >14 (female) | 1.15 |
| <=14 (female) | 0.97 |
| >21 (male) | 1.13 |
| <=21 (male) | 0.95 |
| Physical activity (minutes per week) | |
| <120 | 1.20 |
| >= 120 | 0.84 |
| Smoking status | |
| Non-smoker | 0.92 |
| Smoker | 1.31 |

For each individual in the model, combined HRs for each of the four health behaviours were estimated each year based on the values of that individuals’ behaviour using the continuous risk functions. The combined HR was then applied to the underlying general population mortality rate to estimate an adjusted annual probability of dying.

**F.** **Details of utilities analysis**

Health Survey for England 2008 data [11] were used to develop predictive equations for EQ-5D derived utility scores. The outcome variable was the EQ-5D index score. First, the following potential predictor variables were entered into an ordinary least squares (OLS) linear regression model:

- Age
- Gender
- Fruit and vegetables (portions per day) [Fr_Veg]
- Alcohol (units per week)
- Physical activity (minutes per week)
- Smoking status (smoker or non-smoker)

Second, transformed variables (continuous variables squared and cubed) were entered into the model one by one to see if they improved the model. Variables were left in if they produced a β coefficient with a significance level of p<0.05, increased the adjusted R^2^, lowered the Akaike information criterion (AIC) and Bayesian Information Criterion (BIC) and improved correlation between predicted and observed individual-level values. Based on these criteria, the following variables were added to the model:

- Age^2^
- Fr_Veg^2^
- Fr_Veg^3^
- Alcohol^2^
- Alcohol^3^
- Physical activity^2^
- Physical activity^3^

Third, two-way interaction terms between all the original variables were entered into the model one by one to see if they improved the model. Using the same criteria as for transformed variables, the following interaction terms were added to the model:

- Age*Fr_Veg
- Age*Alcohol
- Age*Physical activity

The results of the final regression model are presented below.

Table F1. Coefficients from OLS model of EQ-5D index score

| **EQ5D** | **β coefficient** | **Std. Err.** | **p-value** | **[95% Conf.** | **Interval]** |
| --- | --- | --- | --- | --- | --- |
| Age | -0.003785 | 0.000564 | 0.000 | -0.00489 | -0.00268 |
| Gender (male = 1) | 0.014181 | 0.003862 | 0.000 | 0.00661 | 0.02175 |
| Fruit and veg (portions per day) | 0.020690 | 0.003715 | 0.000 | 0.01341 | 0.02797 |
| Alcohol (units per week) | 0.001564 | 0.000403 | 0.000 | 0.00078 | 0.00235 |
| Smoking status (smoker = 1) | -0.054127 | 0.004656 | 0.000 | -0.06325 | -0.04500 |
| Physical activity (mins per week) | 0.000231 | 0.000019 | 0.000 | 0.00019 | 0.00027 |
| Age^2^ | -0.000004 | 0.000005 | 0.411 | -0.00001 | 0.00001 |
| Fruit and veg^2^ | -0.003305 | 0.000531 | 0.000 | -0.00435 | -0.00226 |
| Fruit and veg^3^ | 0.000112 | 0.000023 | 0.000 | 0.00007 | 0.00016 |
| Alcohol^2^ | -0.000028 | 0.000004 | 0.000 | -0.00004 | -0.00002 |
| Alcohol^3^ | 6.45E-08 | 1.13E-08 | 0.000 | 4.24E-08 | 8.66E-08 |
| Physical activity^2^ | -2.59E-07 | 1.56E-08 | 0.000 | -2.90E-07 | -2.29E-07 |
| Physical activity^3^ | 4.88E-11 | 3.53E-12 | 0.000 | 4.19E-11 | 5.57E-11 |
| Age*Fruit and veg | 0.000049 | 0.000042 | 0.236 | -0.00003 | 0.00013 |
| Age*Alcohol | 1.61E-05 | 6.05E-06 | 0.008 | 4.25E-06 | 2.80E-05 |
| Age*Physical activity | 2.47E-06 | 2.36E-07 | 0.000 | 2.01E-06 | 2.93E-06 |
| _cons | 0.948994 | 0.015604 | 0.000 | 9.18E-01 | 9.80E-01 |

Table F2. Variance-covariance matrix for the OLS model of EQ-5D index score

**References**

1. Epton T, Norman P, Dadzie AS, Harris PR, Webb TL, Sheeran P, Julious SA, Ciravegna F, Brennan A, Meier PS, Naughton D, Petroxzi A, Kruger J, Shah I: **A theory-based online health behaviour intervention for new university students (U@Uni): Results from a randomised controlled trial.** *BMC Public Health* 2014, **14**:563

2. **Applications (choices) and accepted applicants by institution, 2012 cycle** [http://www.ucas.ac.uk/about_us/stat_services/stats_online/data_tables/heinstitution/2012 (accessed 29 May 2013)]

3 **The R project for statistical computing** [http://www.rproject.org/(accessed 29^th^ May 2013)

4. Curtis L: **Unit Costs of Health and Social Care 2012**. In*.* Edited by Curtis CbL. Canterbury, Kent: Personal Social Services Research Unit 2012.

5. Department of Health: **NHS reference costs: financial year 2011 to 2012**. In*.*; 2012. Available from: https://www.gov.uk/government/publications/nhs-reference-costs-financial-year-2011-to-2012 (accessed 29 May 2013).

6. Oakley JE: **Eliciting Univariate Probability Distributions**. In*.*; 2010. Available from: http://www.jeremy-oakley.staff.shef.ac.uk/Oakley_elicitation.pdf (accessed 30 May 2013).

7. O'Hagan A, Buck CE, Daneshkhah A, Eiser JR, Garthwaite PH, Jenkinson DJ, Oakley JE, Rakow T: **Uncertain Judgements: Eliciting Experts' Probabilities**. Chichester: John Wiley & Sons Ltd.; 2006.

8. **MATCH Uncertainty Elicitation Tool** [http://optics.eee.nottingham.ac.uk/match/uncertainty.php (accessed 31 May 2013)]

9. Gore SM: **Biostatistics and the Medical Research council**. *Medical Research Council News* 1987.

10. Kvaavik E, Batty D, Ursin G, Huxley R, Gale CR: **Influence of individual and combined health behaviours on total and cause-specific mortality in men and women**. *Arch Intern Med* 2010, **170**(8):711-718.

11. National Centre for Social Research: **Health Survey for England 2008**. In*.*; 2008. Available from http://discover.ukdataservice.ac.uk/catalogue?sn=6397 (accessed 29 May 2013).
